# A Simulation Model for External Cephalic Version

**DOI:** 10.7759/cureus.12382

**Published:** 2020-12-30

**Authors:** Carla Baaklini, Natalie Menassa, Jalen T Larios, Derek A Ballas

**Affiliations:** 1 Medicine, Northeast Ohio Medical University, Rootstown, USA; 2 Medicine, University of Akron, Akron, USA; 3 Obstetrics and Gynecology, Summa Health System, Akron, USA

**Keywords:** external cephalic version, simulation, training model, education

## Abstract

Breech presentation complicates as many as 4% of all deliveries. External cephalic version (ECV) is a procedure that involves the external rotation of the fetus through the mother’s abdomen from a breech position into a cephalic position. It provides a beneficial alternative to cesarean section (CS) as it is less invasive, more cost-effective, and mitigates many of the maternal health risks associated with CS. Though ECV has become more widely used in recent years, studies have shown that a large percentage of residency programs lack proper training pertaining to ECV, increasing the need for additional educational intervention. A well-supported method of procedural training that has demonstrated efficacy among trainees is the incorporation of simulation models. While many models have already been developed for various obstetrical procedures, few easily reproducible models currently exist for ECV. The purpose of this study was to develop a reconstructible ECV model that could be utilized for practice by trainees in the field of obstetrics. This study’s proposed ECV model along with a lecture that was presented to residents and data on the effectiveness of the model and comfort with performing the procedure was collected and analyzed. The results demonstrated that when compared to baseline prior to training, levels of comfort with performing an ECV increased amongst trainees after practicing on the model.

## Introduction

Approximately 4% of all deliveries are complicated by a fetal breech presentation at term, posing significantly increased morbidity and mortality rates. Breech babies are most commonly delivered via cesarean section (CS), however, another technique known as an external cephalic version (ECV) can be utilized to reduce fetal breech presentation by externally rotating a fetus through the mother’s abdomen into cephalic presentation [1]. Use of ECV in the case of a breech fetus is highly advantageous as it can help to decrease the necessity for cesarean section and increase the chances of normal vaginal delivery, resulting in an average cost savings of $2462 in the successful completion of this procedure [2]. The risks associated with ECV are minimal, with the most common being abnormal baby heart rate, occurring ∼6% of the time. The remainder of the risks, including the persistence of abnormal baby heart rate patterns, vaginal bleeding, placental abruption, emergency CS, and perinatal mortality, occur less than 1% of the time [1]. With all this considered, the practice of ECV has gained more prevalence in the United States and other countries since the 1980s [3,4], and there is widespread support among various obstetrics and gynecology residency programs for the use and teaching of ECV [5]. Despite this, a survey of these residency programs found that only 65% of the respondents received training in ECV [5]. This, along with the increasing popularity of the procedure, makes it imperative for ECV to be incorporated into the training regimen of obstetricians.

Multiple studies have proven there is an association between the use of simulation models in residency training and performance success, increased comfort level, and decreased time to perform the skill being practiced [6]. Conducting simulations in training have been shown to significantly increase confidence among experienced obstetrician-gynecologists in performing those simulated procedures [7], and have also been previously denoted by obstetricians to be their preferred method of procedural training due to the ability to obtain hands-on experience in a low-risk environment [6]. Creating affordable reconstructible simulation models can be an invaluable way to further strengthen and broaden the skill sets of healthcare workers, which is instrumental in improving patient safety [8]. Simulation models currently exist for ECV and other various obstetric procedures, but our literature review has shown a lack of easily reproducible models for ECV [9]. Therefore, research is necessary to develop an ECV simulation model for cases of fetal malpresentation, as it will be beneficial to future obstetrical training and patient outcomes.

## Technical report

**Figure 1:**
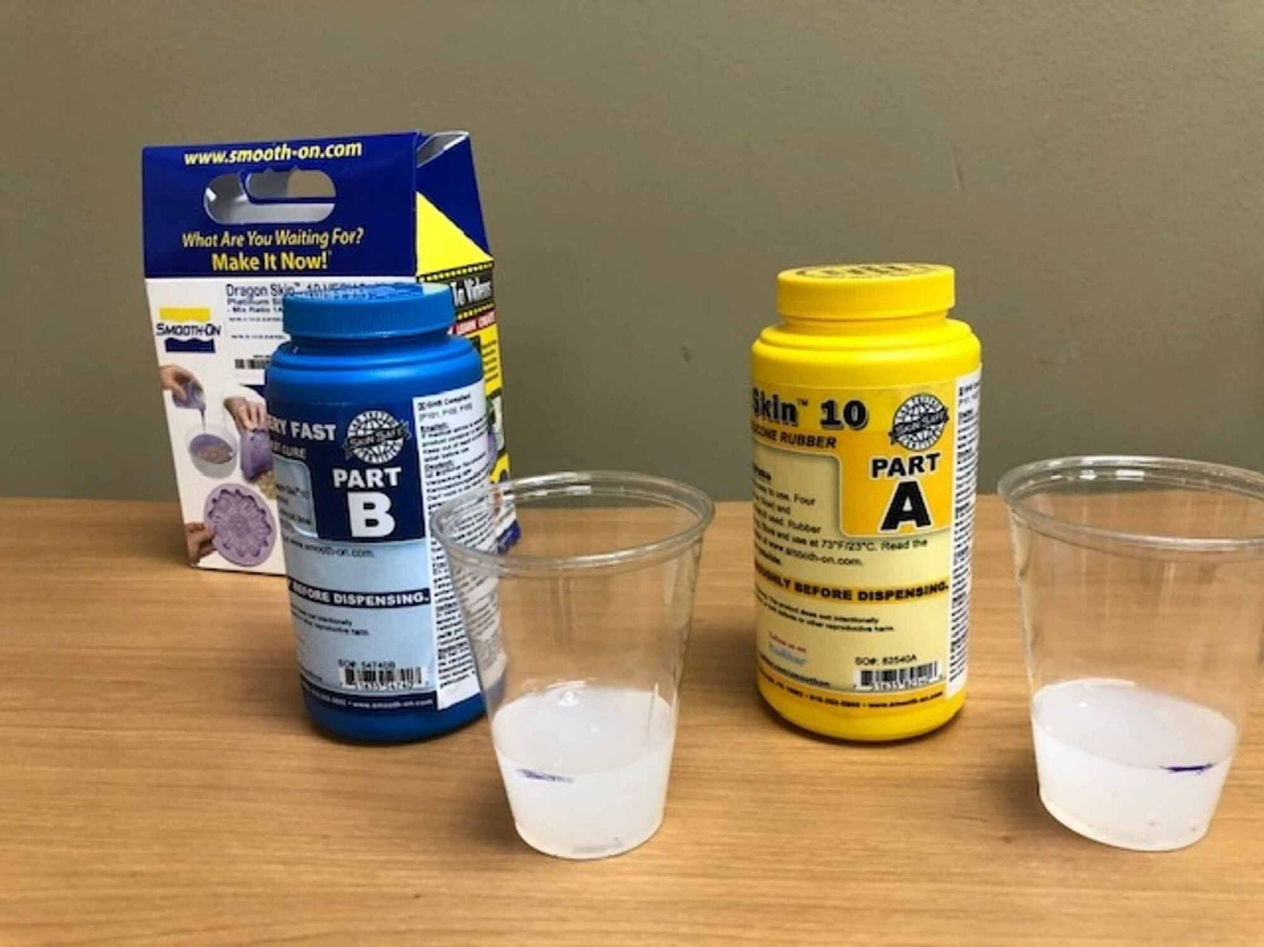
Dragon Skin Smooth-On Silicone™

**Figure 2:**
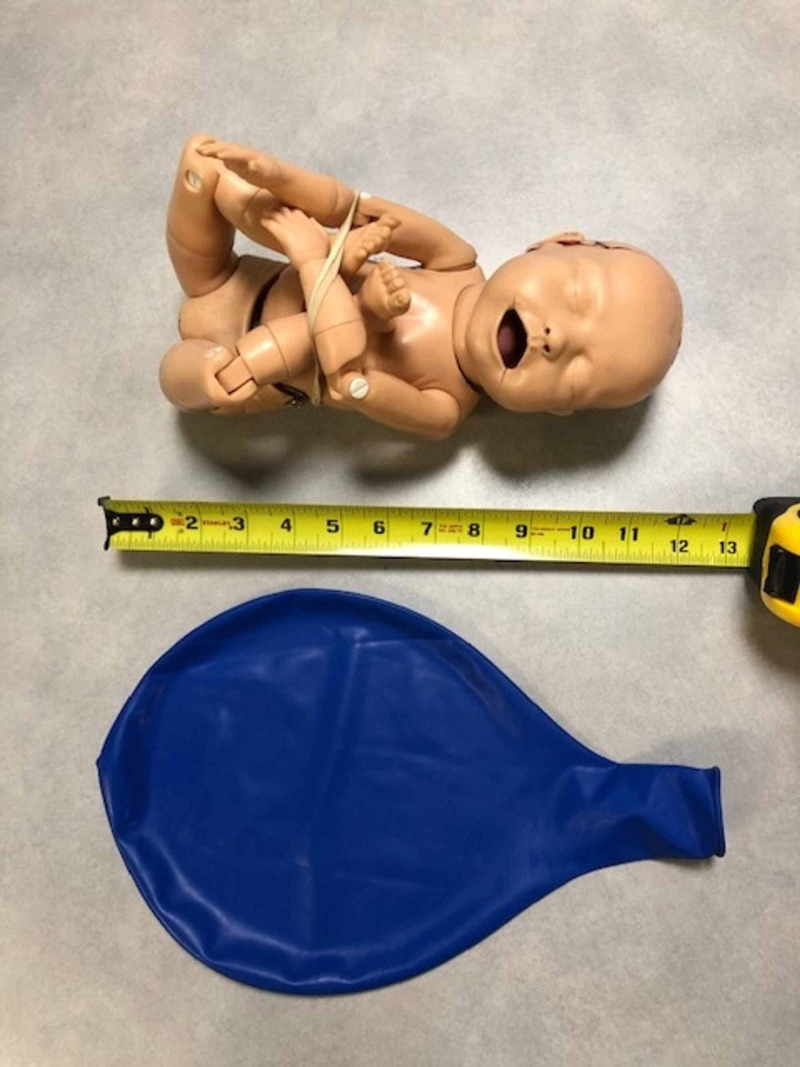
Model baby and 7-inch diameter balloon

**Figure 3:**
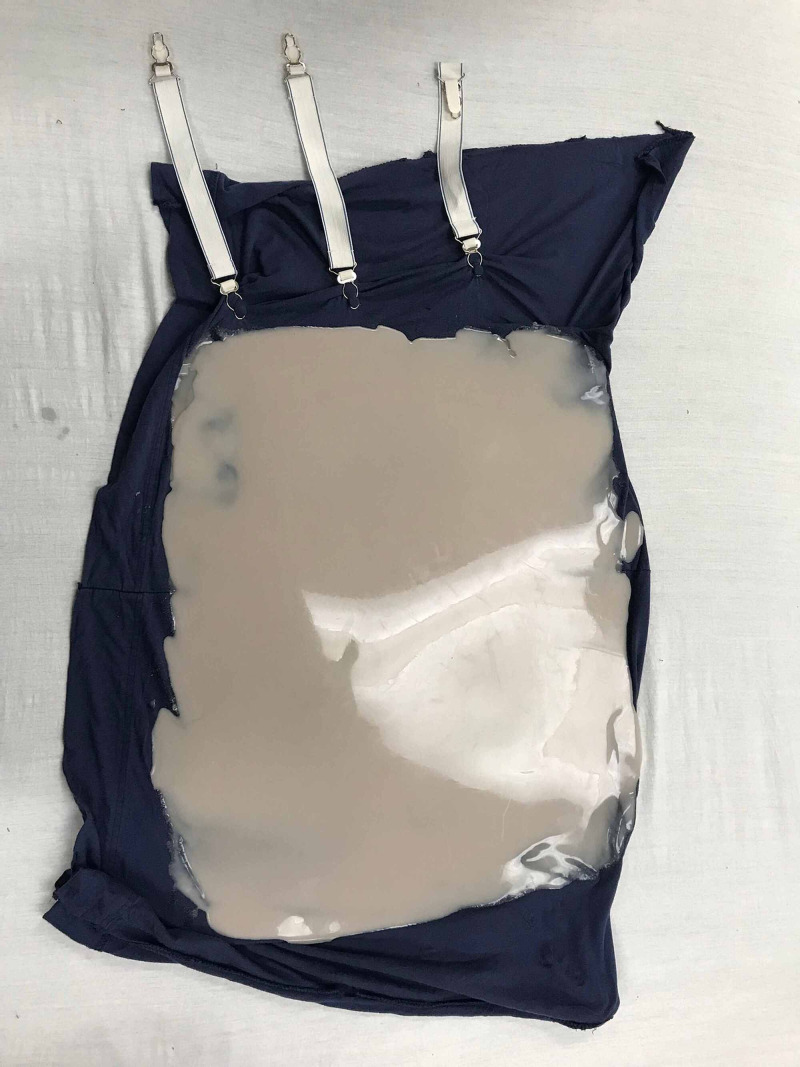
Model abdomen: pillowcase with applied Dragon Skin™ and mattress straps attached

The materials used in the development of the model for the external cephalic version were determined by cost-effectiveness, reproducibility, and practicality. A model of an intrauterine pregnancy was constructed using a pillowcase, mattress straps, Dragon Skin Smooth-On silicone™ (Smooth-On, Macungie, PA; Figure 1), three 7-inch diameter latex balloons, and a model infant (Figure 2). The pillowcase was cut along the seam to create a flat sheet and was used as the base layer of the model abdomen. Three layers of Smooth-On Dragon Skin silicone™ were applied onto the front of the pillowcase until the desired thickness and surface area was achieved (Figure 3). To best replicate human skin and give the model a more realistic appearance, coloring using Smooth-On Silc Pig™ was applied with a toothpick during the mixing of each silicone layer before application. Each layer of Smooth-On silicone™ was applied using tongue depressors. The hardening time for each layer was approximately 30 minutes.

**Figure 4:**
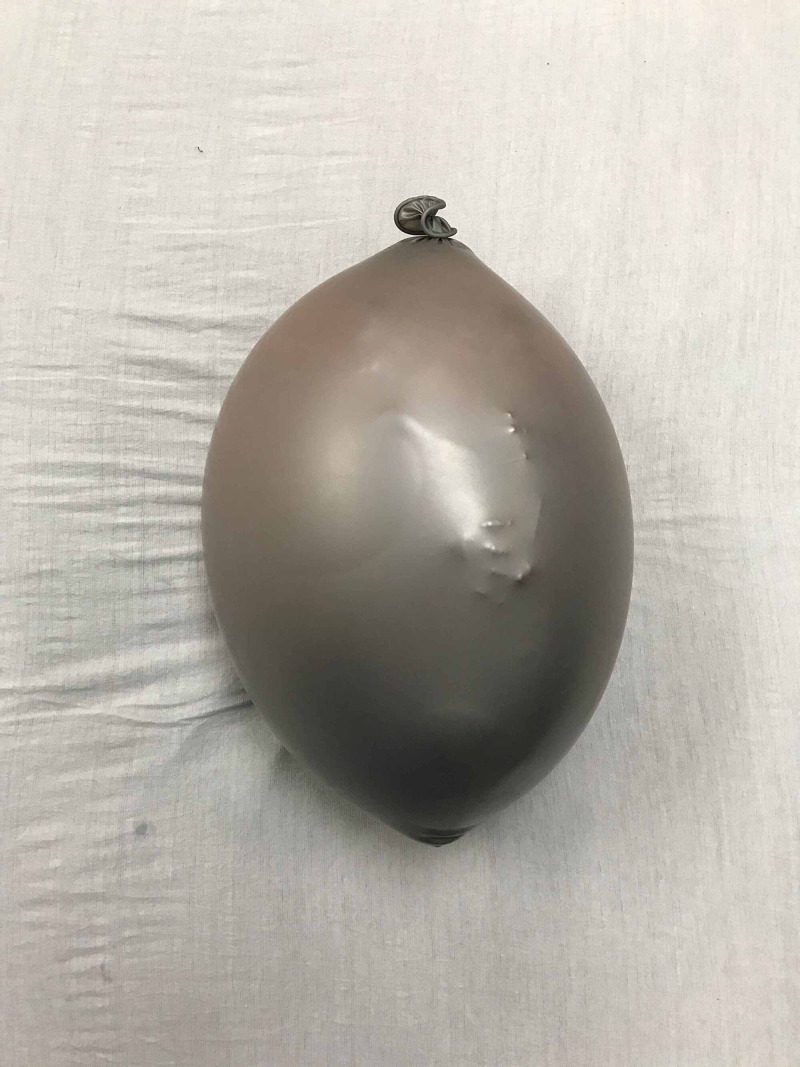
Model uterus: model baby within three balloons

**Figure 5:**
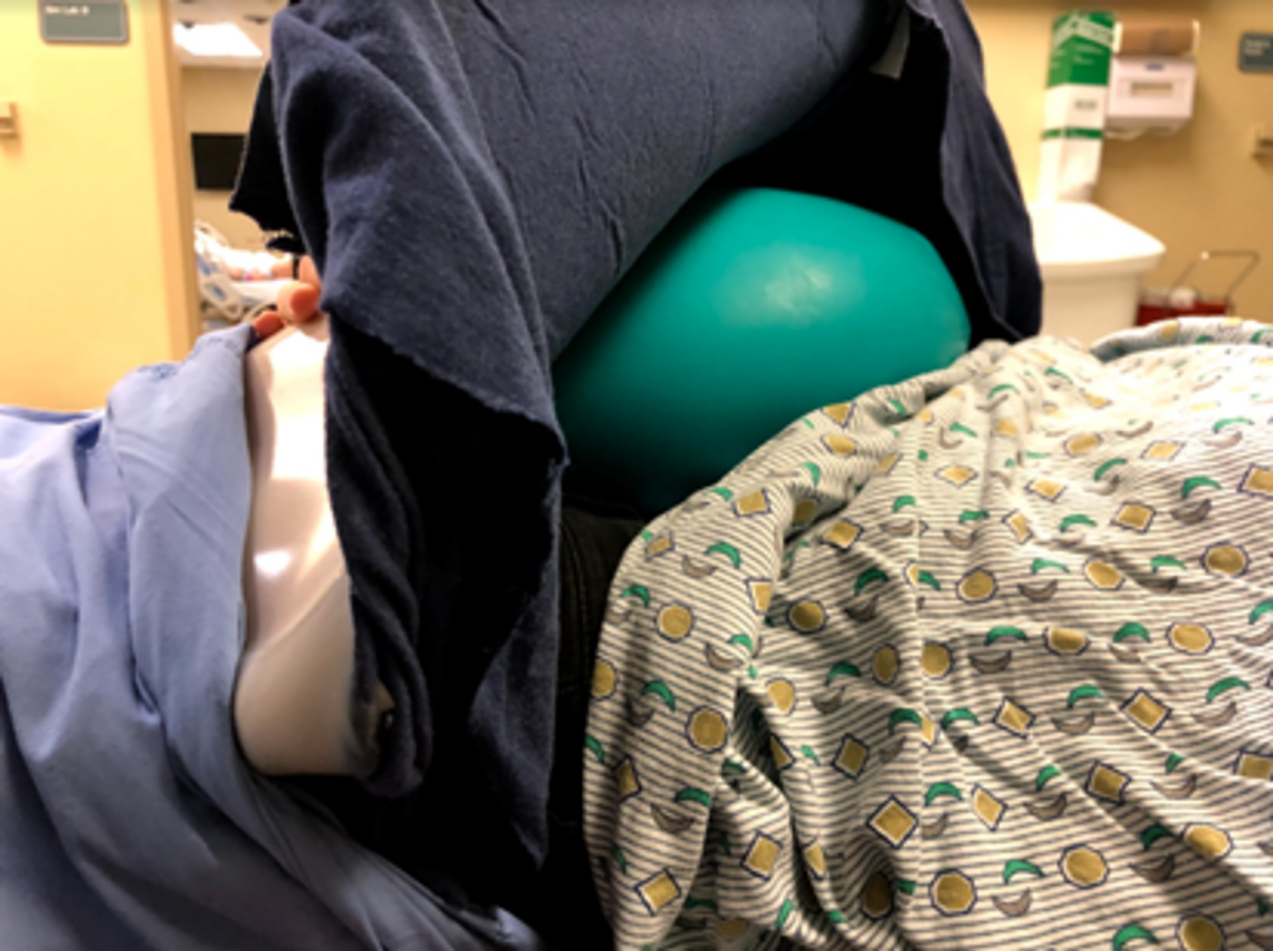
Balloon placed under model abdomen on human volunteer

**Figure 6:**
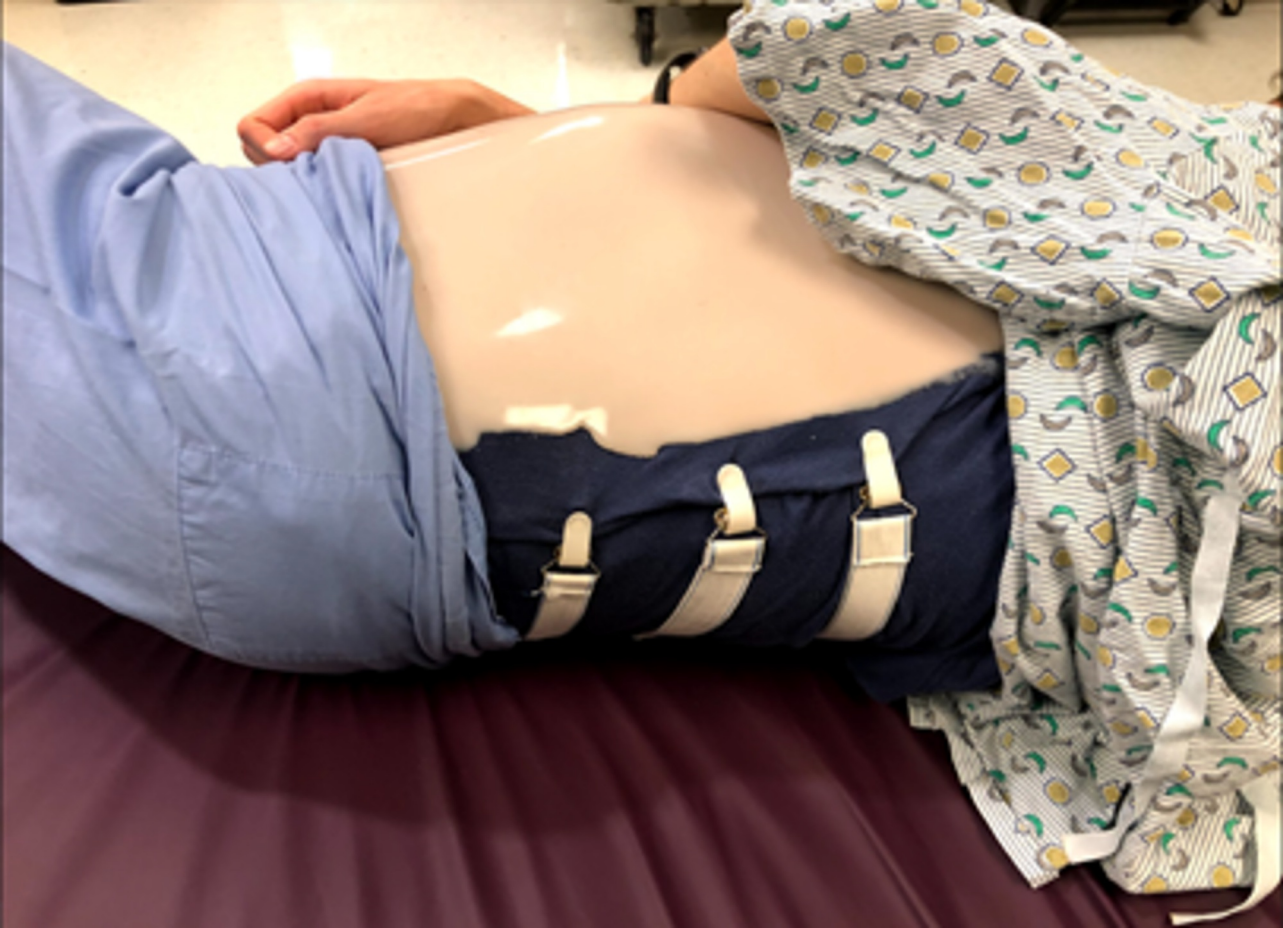
Model abdomen strapped around human volunteer

The three 7-inch diameter latex balloons were used to represent the uterus. The model infant was inserted into the first balloon by stretching the opening over the model infant, headfirst. This step was completed by two people, one holding the balloon outstretched and one inserting the baby into the balloon. The first balloon was then tied off. This allowed for the positioning of the baby in various breech presentations. This was then repeated twice with two additional balloons until the model infant was ultimately surrounded by three balloons in total. The second balloon surrounding the infant was filled with water until the balloon-surrounded model infant was floating and sufficiently covered by water. The third outermost balloon acted as a supportive layer to retain the shape of the model and increase durability to prevent balloon rupture. Excess air was expelled from the balloon and the openings of the two remaining balloons were each knotted off (Figure 4). The model was made complete by placing the model balloon uterus underneath the pillowcase with the simulated skin layers to represent an intrauterine pregnancy (Figure 5). Three mattress straps were attached to the long edges of the pillowcase so that the abdomen-uterus model could be attached around a human volunteer’s abdomen for simulation (Figure 6). The approximate total time to construct and assemble the model was three hours for the pillowcase-model abdomen and 30 minutes for the balloon-model uterus. The approximate total cost to create the abdomen-uterus model was $100.

**Figure 7:**
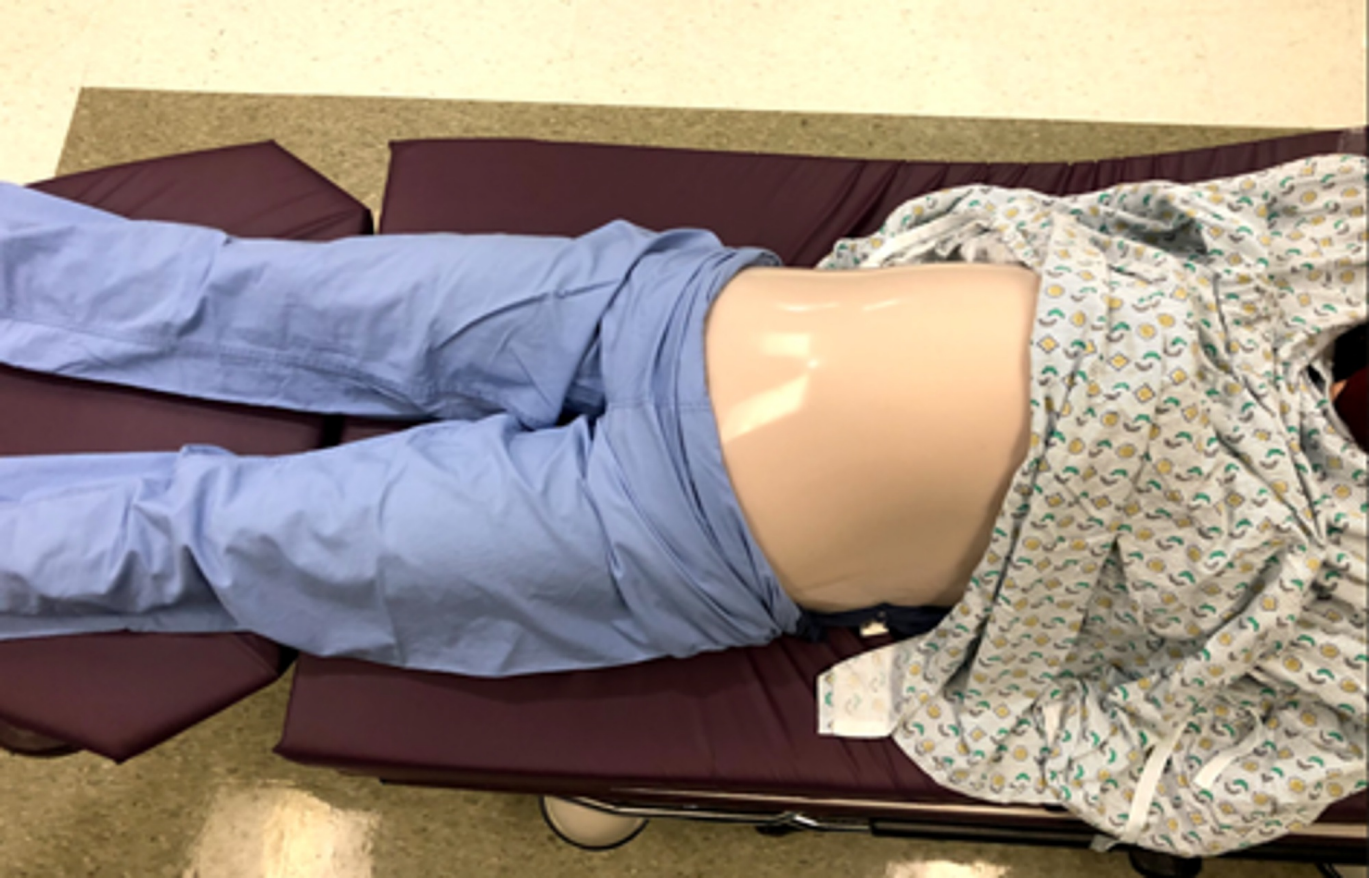
Simulated pregnant patient model

**Figure 8:**
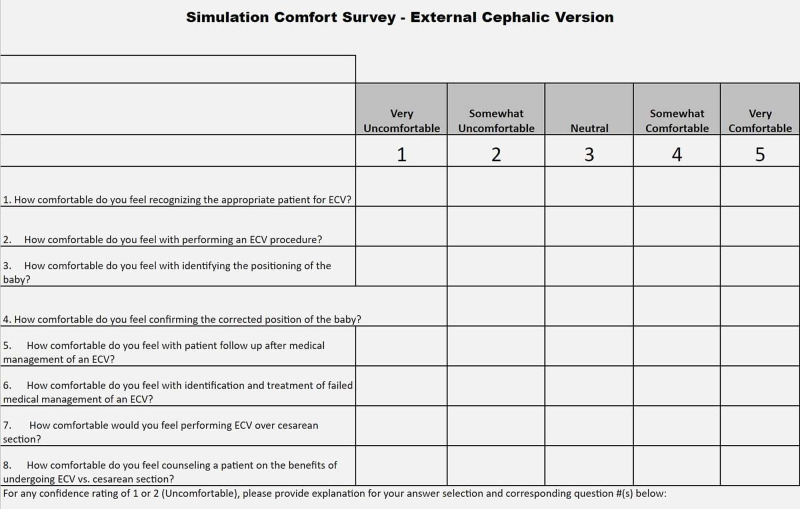
List of questions utilized to assess comfortability in both pre- and post-surveys

An external cephalic version simulation was conducted using the model among OB/GYN residents at various stages in their education. The pillowcase-uterus model was placed around a human volunteer to simulate a pregnant patient who was in need of an ECV. Prior to using the model, residents were first given a lecture on ECV with important information such as instructions on how to conduct the procedure, important statistics about procedure outcomes, how best to counsel patients on the risks and benefits of the procedure, how to determine when ECV is necessary, and other considerable alternatives to the procedure. After the lecture, residents were given the opportunity to attempt an ECV on the simulated pregnant patient model (Figure 7). Optional pre- and post-surveys were collected from the residents via SurveyMonkey^Ⓡ^ to get feedback on their experience with the model, and to measure how comfortable they felt with ECV before and after the simulation (Figure 8).

Results

Eighteen obstetrics and gynecology resident physicians in postgraduate year one (PGY-1) to postgraduate year four (PGY-4) participated in the lecture session followed by an attempt to practice ECV on the simulated pregnant patient model. Out of the 18 resident physicians, 8 (44.44%) had previously performed an ECV while 10 (55.55%) had not. A greater number of individuals, 15 (83.33%), had observed an ECV performed prior to the training experience. 

The number of participants from the pre- and post-training survey decreased from 18 to 11. Additional feedback on the model was collected in the post-training survey that was not asked in the pre-training survey. All of the residents stated that the model was either very effective (72.73%) or somewhat effective (27.27%) and they all stated that they would use the model for further training on ECV. All of the residents were also either very likely (54.55%) or likely (45.45%) to recommend the model to be used to train other medical professionals. 

The residents either strongly agreed (81.82%) or agreed (18.18%) that the material presented in the lecture was helpful and easy to understand. There was greater variation in responses to the question on the residents’ view on how realistic the model was: 18.18% extremely realistic, 45.45% very realistic, and 36.36% somewhat realistic. Most of the residents (90.91%) strongly agreed that the hands-on experience provided by the model gave an understanding of the ECV maneuver that they would not have obtained through lecture alone.

**Figure 9:**
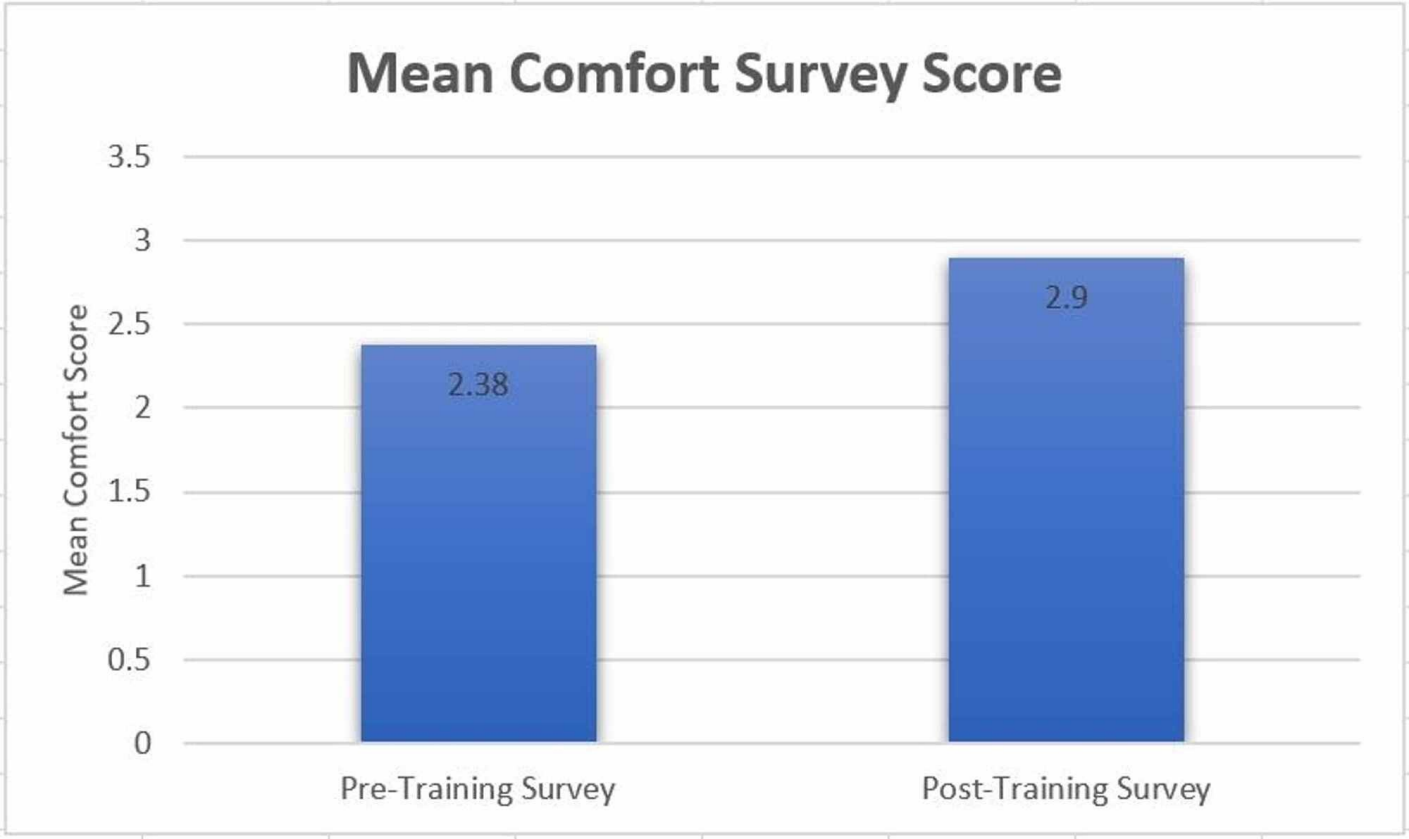
Comfort survey scores

The data from the Likert scale comfort questions in the survey were converted into the numerical score (0=very uncomfortable, 1=somewhat uncomfortable, 2=neutral, 3=somewhat comfortable, 4= very comfortable). The mean comfort score values from the pre- and post-surveys were 2.38 and 2.90, respectively (Figure 9). The standard deviation of the pre- and post-training surveys were 1.07 and 0.74, respectively. An unpaired t-test assuming unequal variances was conducted on these data from the numerically coded Likert scale comfort questions. The two-tailed p-value calculated was 0.000023 which is less than the common level of 0.05. Therefore, the difference between resident physicians' pre- and post-training comfort scores was determined to be significant.

## Discussion

Our model demonstrated a significant increase in residents’ comfort in both recognizing and performing an ECV after participating in our simulation. It is important for residents to have the capability to opt for ECV, should they feel it is an appropriate course of action, instead of resorting only to CS when deciding how to best proceed in the scenario that they encounter breech fetal presentations. Increased knowledge and performance of ECV among residents may help protect against the adverse effects associated with CS, such as the heightened risk for a subsequent pregnancy and fertility complications as well as increased likelihood for asthma and obesity development in the child [10].

A strength of this study is how it expands the breadth of skills and knowledge involved in the performance of obstetrical procedures. Our model allows for increased exposure and practice with ECV, which is typically a less common focus of resident training in obstetrics [5]. This hands-on experience offered in a low-stress environment allows for residents to practice their skills in a meaningful way and gain a deeper understanding of what to expect when they perform an ECV on a real patient for the first time, which increases the potential for success in their performance of the procedure.

Limitations of this study include not having a live ultrasound and fetal heart rate included in the simulation experience, as these are two components that must be monitored continuously in real-time to ensure successful procedure outcomes with human patients. Additionally, the sample size we used to obtain our survey data was not large enough to make the results fully representative of the population of OB/GYN residents around the nation. Furthermore, we only had 11 residents complete our post-survey compared to the 18 who completed our pre-survey, which introduced bias into our survey results. Our simulation was also conducted within a small timeframe, no more than an hour due to the residents' schedules, that prevented each resident from being able to spend a considerable amount of time with the model.

Future studies can expand upon our existing work by creating follow-up measures. This would be beneficial as it would give a more comprehensive assessment of the effects the model has on ECV outcomes over time. Additionally, assessing knowledge and skill measures obtained from the use of the model, rather than comfort, maybe a more objective way of determining how the simulation experience improved resident performance of ECV. Since this model is designed to be on a live patient, it also provides the opportunity for teaching faculty to evaluate the consent and counseling process of the trainee.

## Conclusions

This study sought to address the lack of ECV training and education in residency programs through the creation of an easily reproducible model. Providing an opportunity for simulation training allows for hands-on exposure to procedures and skill improvement while in a low risk and low-pressure environment. The results from this study demonstrated that this intervention was successful in increasing levels of comfort with ECV performance among residents, suggesting this to be a potentially effective teaching model.
